# m7G-related lncRNAs are potential biomarkers for predicting prognosis and immune responses in patients with oral squamous cell carcinoma

**DOI:** 10.3389/fgene.2022.1013312

**Published:** 2022-12-02

**Authors:** Xuefeng Wang, Wei Dong, Yanbo Zhang, Feng Huo

**Affiliations:** ^1^ Department of Stomatology, The Affiliated Hospital of Chengde Medical University, Chengde, China; ^2^ Xiangya Hospital, Central South University, Changsha, China

**Keywords:** oral squamous cell carcinoma, immune signature, treatment, N-7-methylguanosine, lncRNAs

## Abstract

Among head and neck cancers, oral squamous cell carcinoma (OSCC) is the most common malignant tumor. N-7-methylguanosine (m7G) and lncRNAs are both related to the development and progression of tumors. Therefore, this study aims to explore and establish the prognostic signal of OSCC based on m7G-related lncRNAs. In this study, RNA sequencing transcriptome data of OSCC patients were downloaded from The Cancer Genome Atlas (TCGA) database. Therefore, m7G-related lncRNAs were identified as differentially expressed in OSCC. Then, univariate Cox regression analysis and LASSO regression analysis were conducted to evaluate the prognostic significance of differentially expressed lncRNAs. Consequently, the abovementioned lncRNAs were assigned five OSCC patient risk scores, with high-risk and low-risk patients assigned to each group. Different signaling pathways were significantly enriched between the two groups as determined by set enrichment analysis (GSEA). Multivariate Cox regression analysis confirmed the factors used to construct the nomogram model. Then, the prognosis of the nomogram model was evaluated. Consequently, high-risk individuals had higher immune infiltration levels. According to the results of a study that evaluated the sensitivity of different risk subgroups to antitumour drugs, the high-risk group had a high sensitivity to doxorubicin. By performing real-time polymerase chain reaction (RT‒PCR), we verified the expression of these five m7G lncRNAs. Therefore, the model based on five m7G-related lncRNAs was able to predict the overall survival rates of OSCC patients and guide their treatment. It can also spur new ideas about how to prevent and treat OSCC.

## Introduction

Among the various types of head and neck squamous cell carcinoma, oral squamous cell carcinoma (OSCC) is by far the most common (Bray et al., 2018; Chow 2020). Although surgery can be an effective treatment for OSCC, the 5-year survival rate for OSCC is only 60% (Bray et al., 2018). Cancer survival rates are low due to immune escape and tumor metastasis (Parmar et al., 2021). When patients have OSCC with tumor metastases, they usually need neck lymph node dissection, which is more difficult to perform. Postoperative dysfunction is also likely to result from the enlarged surgical area and further affect the prognosis (Romer et al., 2019). It is not possible to detect OSCC or assess metastatic disease early using new techniques, as well as a model to predict the prognosis of patients, is another concern (Mishra 2012; Nikitakis et al., 2018). Therefore, it is a challenge for oral clinicians to reduce the mortality of OSCC by developing new diagnosis and treatment strategies (Khurshid et al., 2018).

Long noncoding RNAs (lncRNAs) are a subset of RNAs that are longer than 200 nucleotides (Spizzo et al., 2012). Despite the fact that lncRNAs do not encode proteins, certain studies have shown that lncRNAs interact with chromatin, protein, and RNA to perform a variety of tasks in cell biology (Begolli et al., 2019). Studies conducted in the past have implicated a number of lncRNAs in the development and outcome of oral squamous cell carcinoma (Li et al., 2017; Huang et al., 2020; Meng et al., 2021). RNA modification plays an important role in the transcription and posttranscriptional regulation of gene expression (Weiße et al., 2020). RNA methylation is a reversible posttranslational modification that affects many biological processes in epigenetics, such as RNA stability, localization, mRNA translation and translocation (Ha et al., 2019). There has been great interest recently in N-7-methylguanosine (m7G), one of the most common RNA modifications (Pandolfini et al., 2019). Recent studies have shown that m7G-related lncRNAs are closely related to the prognosis of tumors (Ming and Wang. 2022; Sun et al., 2022; Zhao et al., 2022). However, there are no reports about m7G-related lncRNAs and OSCC.

Here, our study identified five prognostic risk models associated with lncRNAs related to m7G. Based on the results of this model, it is possible to independently predict the prognosis and survival of OSCC patients. Finally, the potential immunotherapy and drug sensitivity prediction of this model are developed.

## Materials and methods

### Information on oral squamous cell carcinoma

RNA transcriptome data of 404 tumor samples and 54 normal samples were obtained (https://portal.gdc.cancer.gov/), as well as clinical information on patients with OSC. The clinical information included age, sex, race, smoking, alcohol, pathologic stage, stage N, stage T, and tumor grade. To reduce statistical bias, we excluded patients with no survival information or a survival time of less than 3 months from further evaluation.

### Identification of m7G-related lncRNAs

After reading and summarizing the published literature, 23 m7G regulatory factors were identified. The m7G regulatory factors include METTL1, WDR4, NSUN2, DCP2, DCPS, NUDT10, NUDT11, NUDT16, NUDT3, NUDT4, NUDT4B, AGO2, CYFIP1, EIF4E, EIF4E1B, EIF4E2, EIF4E3, GEMIN5, LARP1, NCBP1, NCBP2, NCBP3, EIF3D, EIF4A1, EIF4G3, IFIT5, LSM1, NCBP2 L, and SNUPN (Rong et al., 2022). A total of 16,773 lncRNAs were obtained in the OSCC data set of TCGA. We constructed a coexpression network of m7G methylation regulatory factors and lncRNAs by using the “igraph” package in the R program, and the conditional screening was (Pearson R)>0.3, *p* < 0.001. These lncRNAs are significantly related to the m7G methylation regulator (m7GR) and have been identified as m7G methylation-related lncRNAs.

### Differential expression analysis

Differentially expressed genes (DEGs) were detected between normal and tumor tissue samples by FDR 0.05 and |log2FC| > 2. According to the descending value of |log2FC|, the expression data of upregulated DEGs and downregulated DEGs were used, and R 4.1.2 was used to perform two-way hierarchical clustering analysis.

### Analyses of univariate cox regression and LASSO regression

To identify lncRNAs with prognostic significance, univariate Cox regression analysis was performed on tumor samples and the control group. By using LASSO regression, a m7G-related lncRNA prognostic model (m7G-LncM) for OSCC patients was constructed using the R package glmnet. The steps for calculating the LASSO regression model’s risk score are as follows: $\text{Risksore}=\sum_{\text{i}=1}^{\text{n}}\beta_{\text{i}}\times {Ε}_{\text{i}}$

Ei is the expression value of the i gene in the model, and *β*i is the coefficient calculated by LASSO. According to this equation, we calculated the risk score of each OSCC patient. Patients were divided into high-risk and low-risk groups based on their median risk scores. Risk scores were compared to the overall survival rate (OS) at 1, 3 and 5 years using the receiver operating characteristic (ROC) curve, which was used to determine the area under the curve (AUC). To further demonstrate that the risk model of m7G-related lncRNAs has the ability to distinguish tumor patients, the “Limma” and “scatterplot3D” packages in R were used to conduct principal component analyses (PCA) on the genes associated with risk and the lncRNAs associated with m7G.

### An oral squamous cell carcinoma predictive nomogram

An independent prognostic factor included in the nomogram was screened using univariate and multivariate Cox regression (Wang et al., 2021). In accordance with the results of the univariate and multivariate Cox regressions, the probability of 1-, 3- and 5-year OS of OSC patients was evaluated with the “RMS” R package. The C-index and calibration curve were used to assess the nomogram’s capacity for prediction and discrimination. Decision curve analysis (DCA) was used to evaluate the net benefits of patients with OSCC under different threshold probabilities. The completion of DCA depends on two packages (stdca and dca).

### Immune cell infiltration

For the score calculation of immune cell infiltration, ssGSEA was carried out with the gsva software package to calculate immune cell scores and assess immune-related pathways (*p* < 0.05) (Chen et al., 2022).

### Mutation burden analysis

After obtaining the nucleotide variation data of OSCC, the genetic mutation burden of OSCC samples was analysed by Perl software. In OSCC patients with elevated and low risks for tumor mutation, the “limma” software package was used to analyze the difference in tumor mutation burden. Last but not least, all OSCC patients were grouped according to tumor mutation burdens and survival analysis was combined with the previously analysed high- and low-risk groups. With the survival and survminer R packages, we conducted survival analysis of mutation burden analysis.

### Gene set enrichment analysis

To explain the differential signaling pathways between the high-risk group and the low-risk group of OSCC patients, we further understood the biological functions of these pathways. The method used in this study was Kyoto Encyclopedia of Genes and Genomes (KEGG) pathway analysis.

### Analysis of oral squamous cell carcinoma immunotherapy and screening of potential drugs

Based on the large-scale drug screening data in the database of cancer drug sensitivity genomics (GDSC) (https://cancerrxgene.org), this study combined with this genome analysis is beneficial to systematically identify the tumor response to chemotherapy drugs. In addition to calculating the maximum inhibitory concentration (IC50) of commonly used chemotherapy drugs for OSCC, an evaluation of its clinical utility was conducted. The R package used in this operation is “pRRophetic.” An analysis of Wilcoxon symbolic rank tests was carried out to compare the differences in IC50 between the high-risk and low-risk groups. For the data result, a block diagram is created by “pRRophetic” and “ggplot2” (Geeleher et al., 2014).

### LncRNAs in oral squamous cell carcinoma tissues analysed using quantitative real-time PCR

This study was conducted with tissues kindly provided by the Department of Stomatology of the Affiliated Hospital of Chengde Medical University. Medical ethics approval was obtained for sample acquisition. A total of three nontumor oral tissues and three OSCC tissues were obtained in October 2022 and stored at −80°C. Total RNA was isolated with TRIzol reagent, and quantitative real-time fluorescence quantitative PCR (qRT‒PCR) was performed with primers from Suzhou Genewiz Company and premix from SYBR Master Mix. The results were analysed using the 2−ΔΔCt method, and each sample test was repeated three times. Supplementary Table S1 contains details about the primer sequences.

## Results

### Differentially expressed m7G-related lncRNAs

**FIGURE 1:**
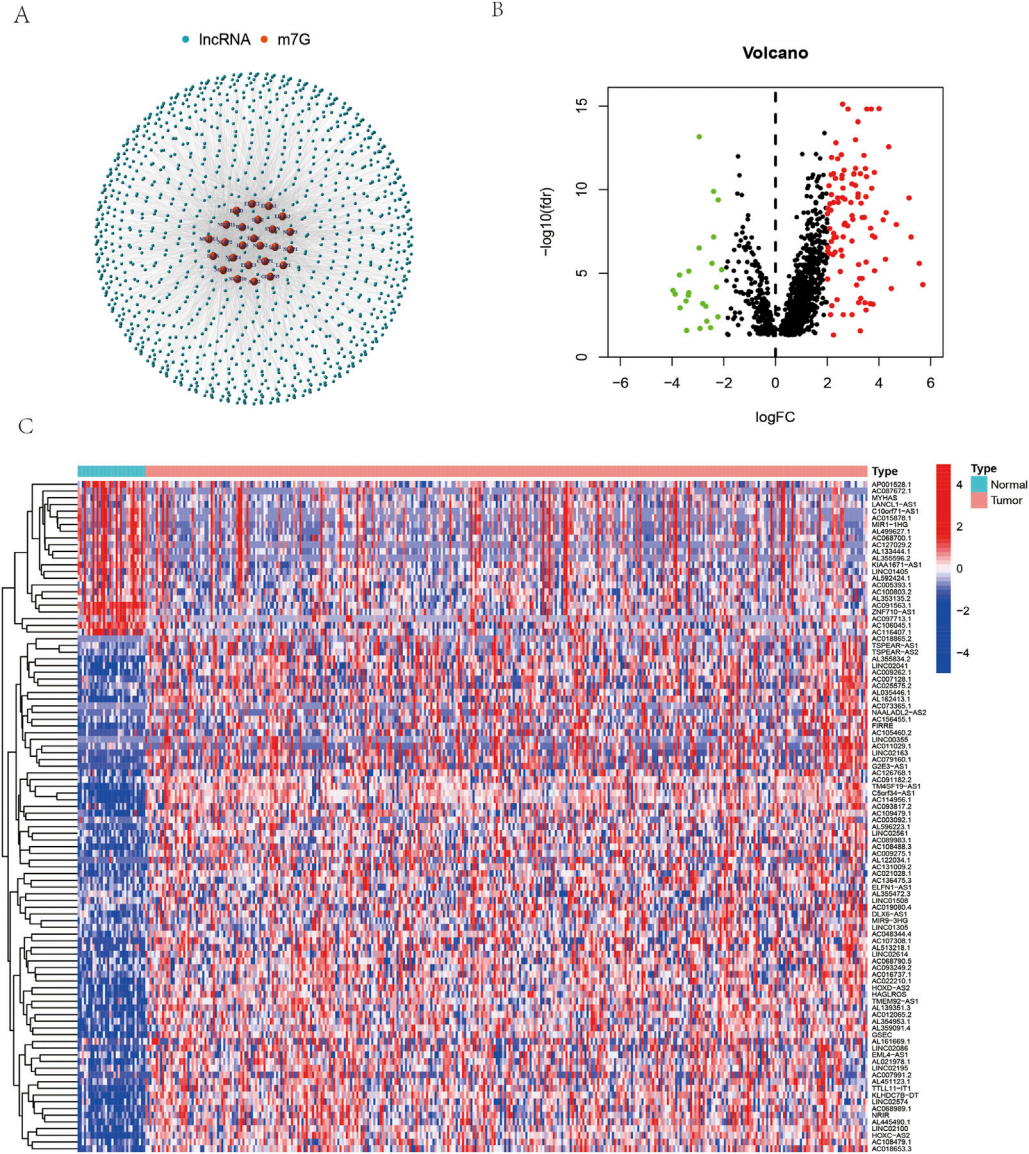
m7G-related lncRNAs in patients with oral squamous cell carcinoma (OSCC). **(A)** The coexpression network map between m7G and lncRNAs in OSCC. **(B)** Volcano plots of differentially expressed m7G-related lncRNAs between normal and tumor tissues. **(C)** Heatmaps of differentially expressed m7G-related lncRNAs between normal and tumor tissues.

First, according to Pearson correlation criteria, m7G-related lncRNAs were screened between 404 OSCC patients and 54 normal tissues. There were 1302 lncRNAs in total. In Figure 1A, the lncRNAs related to the m7G gene are shown as a network diagram. A total of 206 long noncoding RNAs differed in expression between the original cancerous tissue and normal tissue according to a differential gene expression analysis (Figure 1B), of which 36 genes were downregulated and 170 genes were upregulated (Figure 1C).

### Identification of prognostic m7G-related lncRNAs in patients with oral squamous cell carcinoma

**FIGURE 2:**
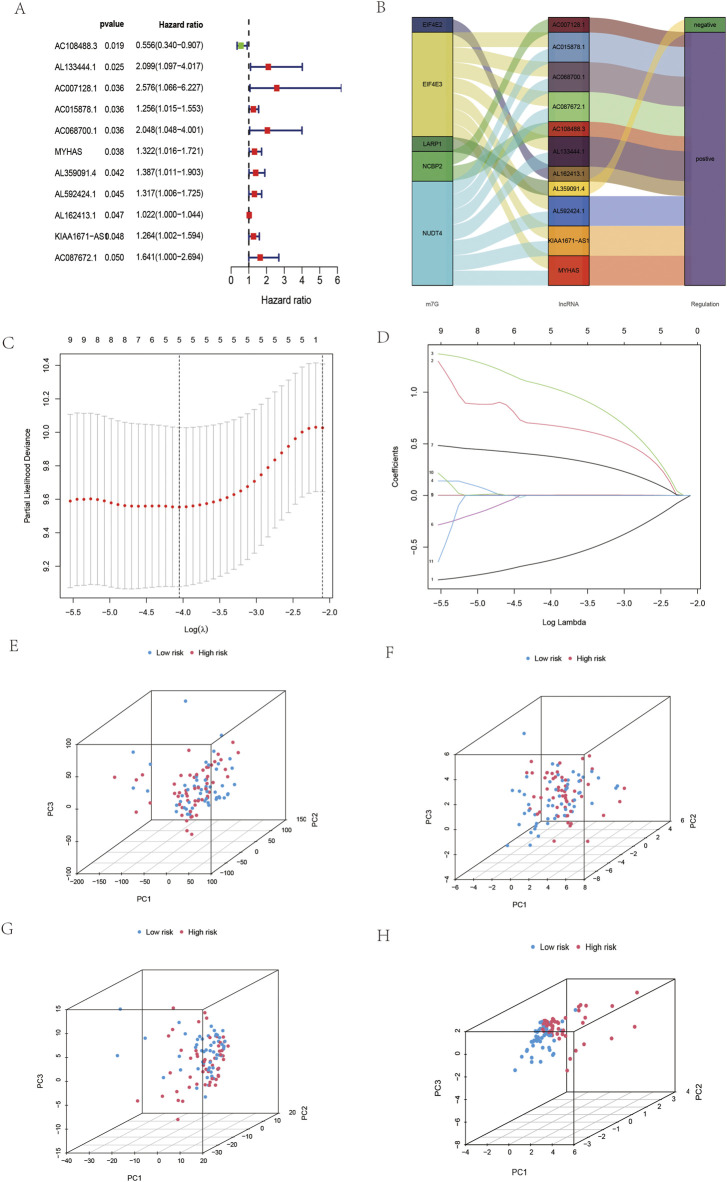
Construction of the five prognostic m7G-related lncRNAs in OSCC. **(A)** Univariate Cox regression screening of m7G-related lncRNAs in OSCC. **(B)** Sankey diagram of OSCC. **(C,D)** Lasso regression was used to obtain five m7G-related lncRNAs in OSCC. **(E)** PCA of all genes in OSCC. **(F)** PCA of m7G-related genes in OSCC. **(G)** PCA of m7G-related lncRNAs in OSCC. **(H)** PCA of five m7G-related lncRNAs in OSCC.

Univariate Cox regression was applied to 206 differentially expressed lncRNAs, and 11 m7G-related lncRNAs were identified (Figure 2A). To demonstrate this network of risk types related to the m7G regulator and lncRNAs, a Sankey diagram was constructed. (Figure 2B). Then, 11 m7G-related lncRNAs were subjected to Lasso regression to obtain five m7G-related lncRNAs. According to five m7G-related lncRNAs (Figures 2C,D), the prediction signal (figure) was constructed, and the risk coefficient of each lncRNA was obtained. The formula calculates the risk score as follows: AC108488.3*(−0.635)+AL133444.1*(0.683)+AC007128.1*(1.056)+AL359091.4* (0.382)+AL162413.1*(0.004). In addition, according to the model of all genes, m7G-related genes, m7G all-related lncRNAs and five m7G-related lncRNAs, PCA was conducted in this study. High-risk vs. low-risk differences among five m7G-related lncRNAs were the most obvious among the five (Figures 2E–H). As a result, the model we built is capable of distinguishing people at high and low risk.

**FIGURE 3:**
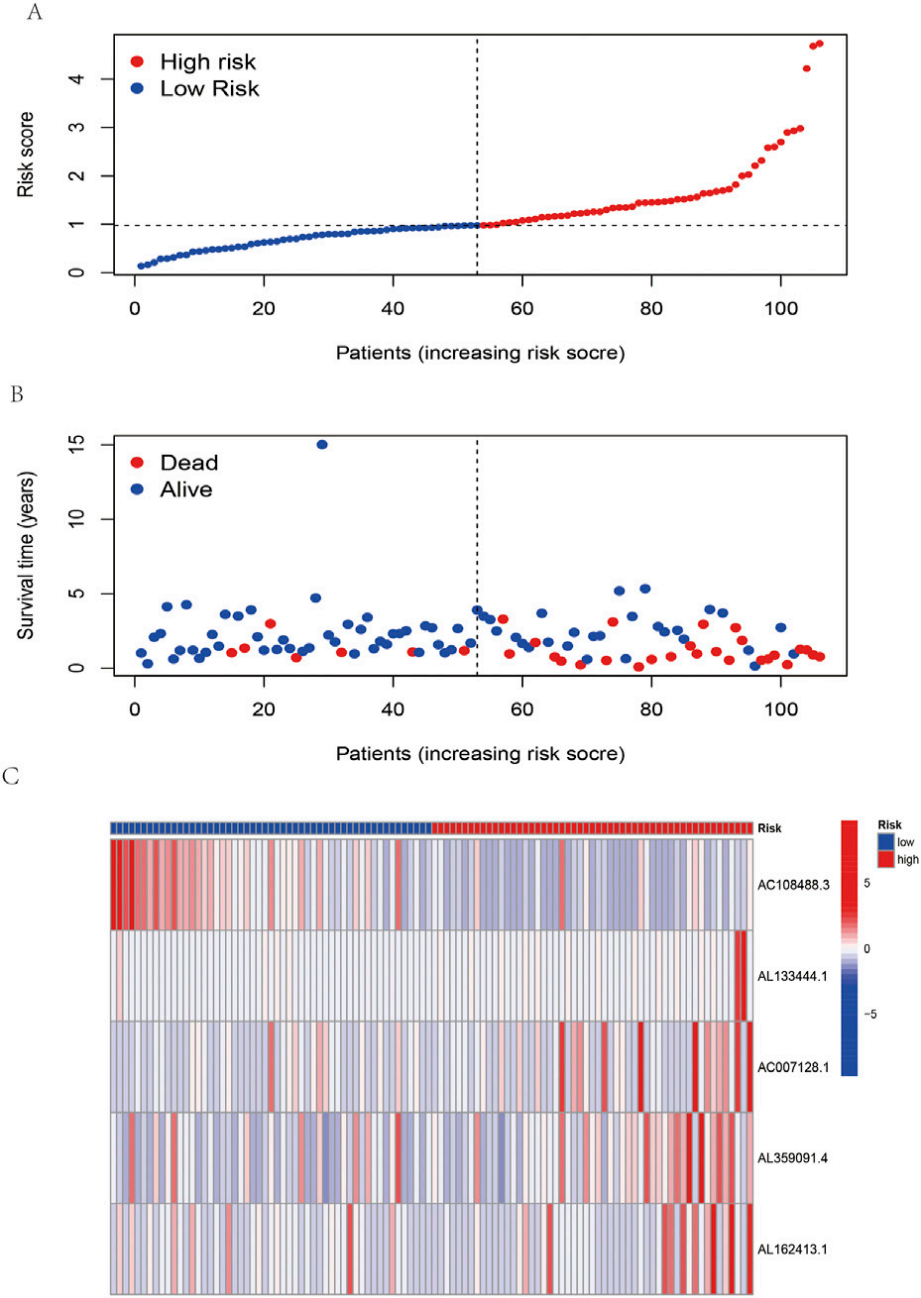
Risk assessment model in patients with OSCC. **(A)** Distribution of OSCC patients based on the risk score. **(B)** The survival status for each patient with OSCC. **(C)** Heatmap of five m7G-related lncRNAs in OSCC.

**FIGURE 4:**
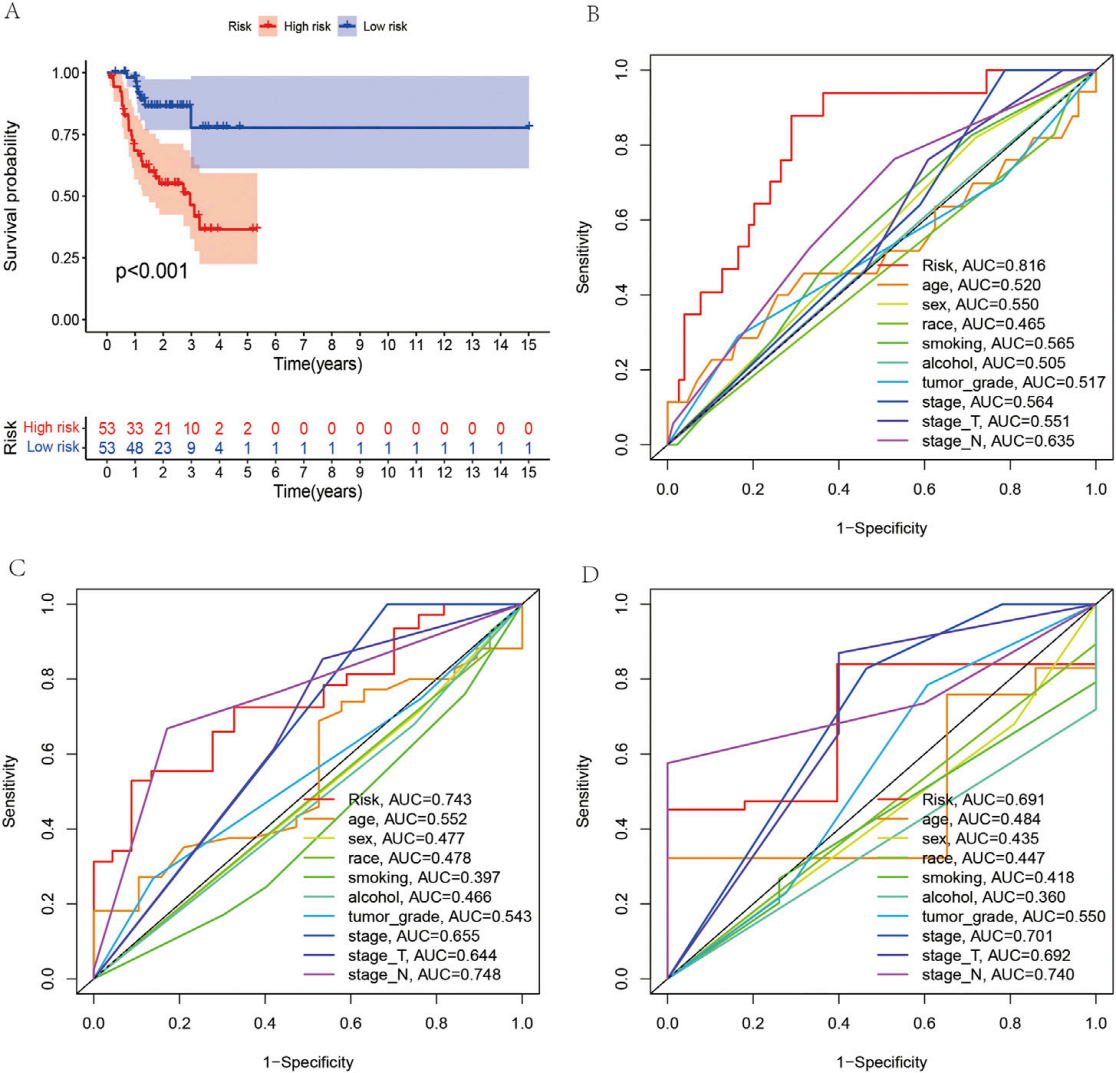
Survival analysis between the high- and low-risk groups and receiver operating characteristic (ROC) curve analysis of clinical and pathological characteristics. **(A)** Survival analysis of all OSCC patients between the high- and low-risk groups. **(B–D)** ROC curves reveal the predictive accuracy of 1-, 3-, and 5-year survival in OSCC.

**FIGURE 5:**
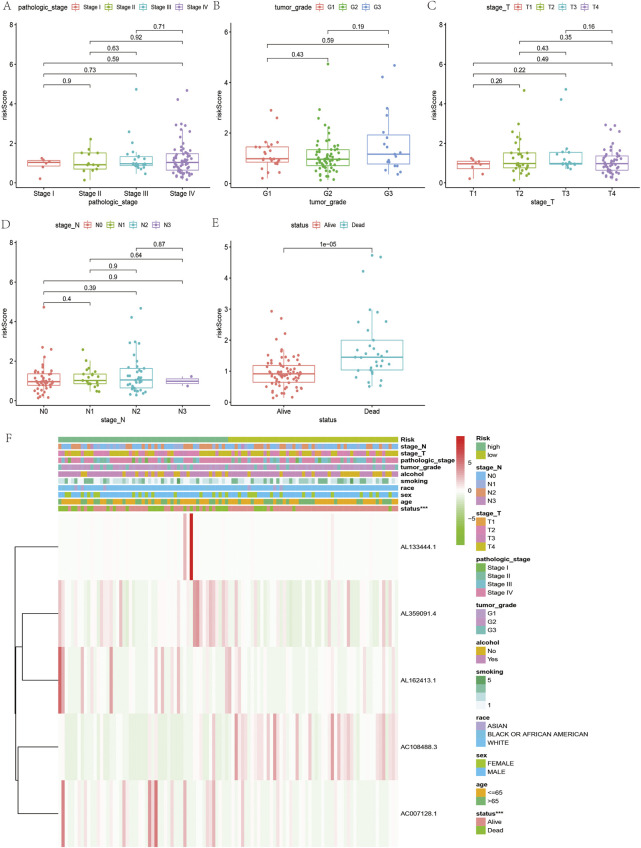
Relationship between clinicopathological features and the risk score in OSCC. **(A–E)** Comparison of risk scores of clinicopathological features in OSCC. **(F)** Heatmap of the five m7G-related lncRNAs in low- and high-risk OSCC.

According to the coefficient value of lncRNAs, it can be judged whether a lncRNA is a risk or a protection. Following the risk score, each OSCC sample from TCGA was divided into two low- and high-risk groups (Figure 3), using Kaplan-Meier survival curves to reflect the relationship between the risk score and survival rate. As shown in Figure 4A, there was a significant difference between the two groups (*p* < 0.001). The risk curve and graph indicate that the number of deaths increases obviously with increasing risk score (Figure 3A). This study showed a good correlation between OSCC survival and risk score. In addition, the multitime ROC curve demonstrated that risk signatures accurately predicted OSCC survival rates at 1, 3, and 5 years (Figures 4B–D). These results indicate that these five m7G-related lncRNAs play an important biological role in the development of OSCC. Additionally, this study compared the clinical characteristics of high- and low-risk patients (Figure 5). The results There was no difference in any kind of clinical information among the different groups, except the status condition.

### Construction and evaluation of the prognostic nomogram

**FIGURE 6:**
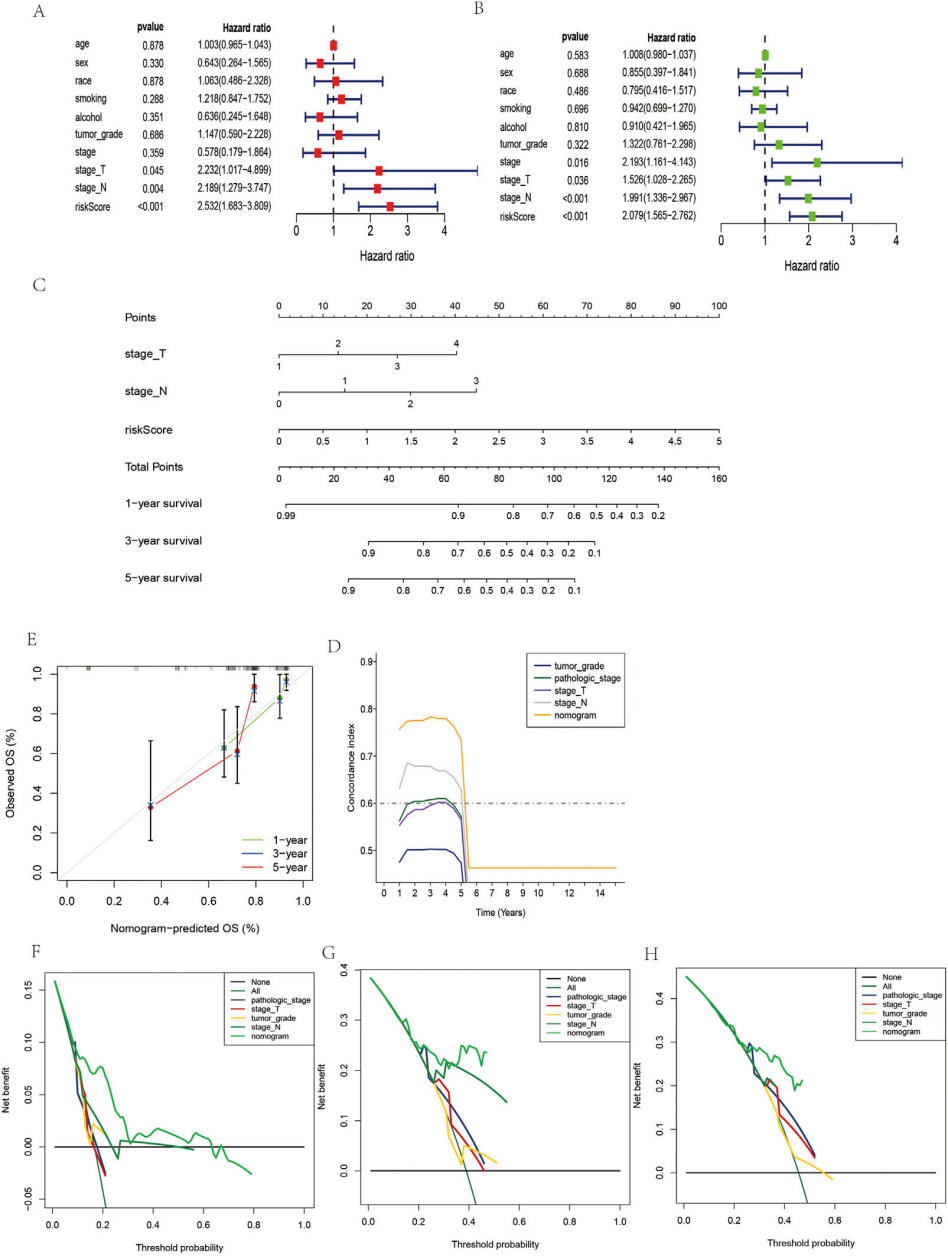
Construction and evaluation of nomogram prediction for the overall survival rate of OSCC patients. **(A)** Univariate Cox regression analyses in OSCC. **(B)** Multivariate Cox regression analyses in OSCC. **(C)** Nomogram prediction of overall survival in OSCC. **(E)** Calibration plots of the nomogram in OSCC. **(D)** C-index of the nomogram and clinical features in OSCC. **(F–H)** Decision curve analyses of the prognostic nomogram and clinical features for 1-, F-, G-, and 5-year **(H)** risk in OSCC.

As shown in Figure 6, considering the clinical information of OSCC patients (including age and sex) and m7G-related lncRNAs, univariate Cox regression (Figure 6A) and multivariate Cox regression (Figure 6B) were performed. Finally, three variables were included in this study as a nomogram to construct prognosis (Figure 6C). The calibration curve’s 1, 3, and 5-year predictions demonstrate that the development of the nomogram has some predictive value for the prognosis of OSCC (Figure 6E). In addition, OSCC prognosis is improved by the C index over other clinical information. In DCA, it can be seen that the nomogram has more clinical value for the prognosis of OSCC patients at 1, 3 and 5 years than other clinical stages (Figures 6F–H).

### Immune cell and immune function enrichment analysis

**FIGURE 7:**
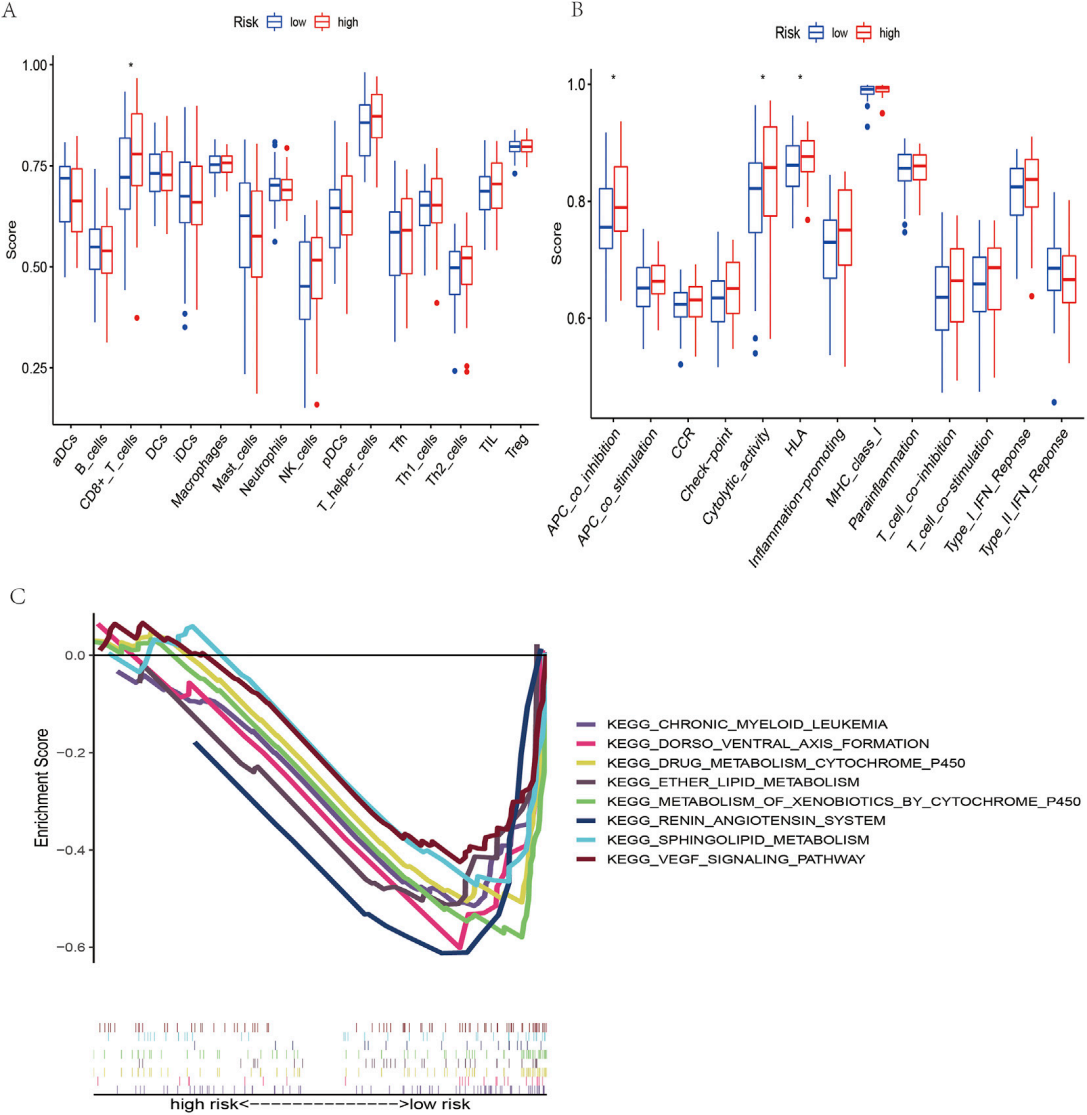
Immune cell infiltration analysis and KEGG pathway analysis. **(A)** Immune cells between the low- and high-risk groups of OSCC patients. **(B)** Immune function between the low- and high-risk groups of OSCC patients. **(C)** KEGG mainly participated in m7G-related lncRNAs in OSCC.

Regarding functional analysis, our study also examined 16 immune cells and 13 immune-related pathways for enrichment fraction and activities between low-risk and high-risk OSCC patients by single-sample genome enrichment analysis (ssGSEA). In patients with OSCC (Figure 7A), it was usually the high-risk subgroup that had high levels of immune cell infiltration, especially CD8^+^ T cells. Low-risk groups had downregulated APC coinhibition, cytolytic activity, and HLA response pathways. (Figure 7B).

### Gene set enrichment analysis

According to the KEGG pathway analysis, the high-risk group did not have an enrichment in the signaling pathway, while chronic myeloid leukemia, dorsoventral axis formation, drug metabolism cytochrome p450 and the VEGF signaling pathway were enriched in the low-risk group (Figure 7C). This indicates that the signaling pathway and its related lncRNAs affect the occurrence, development and prognosis of OSCC, which is a mechanism worthy of further study.

### Analysis of oral squamous cell carcinoma tumor mutation

**FIGURE 8:**
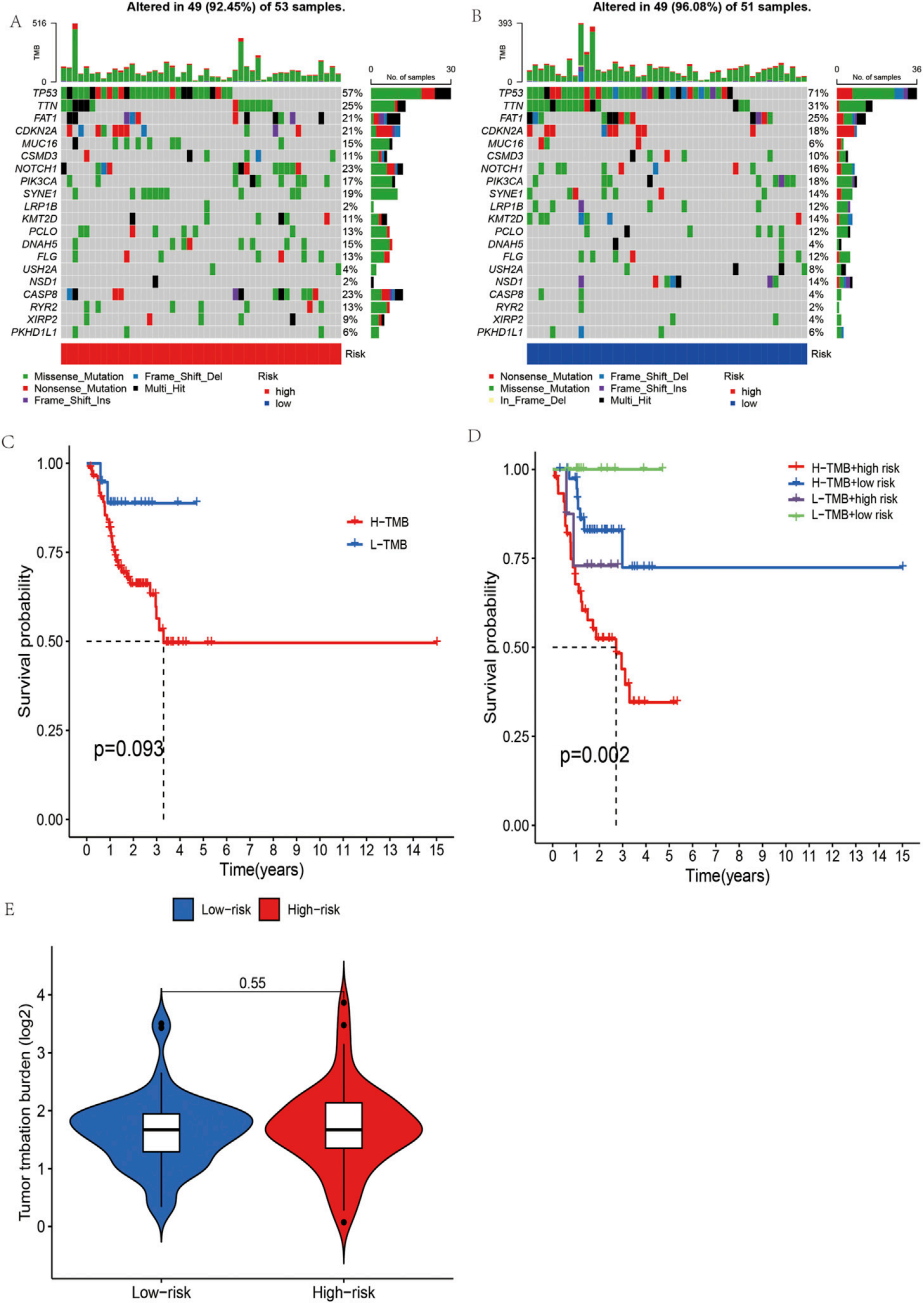
The mutational burden and survival analysis in OSCC based on five m7G-related lncRNAs. **(A,B)** Mutational signals of the high- and low-risk groups in OSCC. **(C,D)** Combined survival analysis of high- and low-mutation groups and high- and low-risk groups. **(E)** Grouped burden differences between high- and low-risk groups.

Our first step in examining mutation differences between high-risk and low-risk groups was to examine mutation rates. The mutation frequency in the high-risk group (92.45%) was lower than that in the low-risk group (96.08%), according to the results (Figures 8A,B).In both groups, the most frequently mutated gene was TP53. In this study, we collected the somatic mutation data of OSCC and calculated the corresponding TMB score to study the potential role of OSCC tumor mutation load. It can be seen in Figure 8E shows that the mutation load in OSCC did not differ significantly between the high- and low-risk groups. With the median as the boundary point, patients were divided into “high TMB” and “low TMB” groups, and the results of survival analysis showed that there was no significant difference between the two groups (Figure 8C). It is possible, however, to determine the combined survival curve by combining tumor mutation load and risk score analysis. An interesting finding was that TMB and risk score were significantly correlated with OSCC survival (Figure 8D).

### Oral squamous cell carcinoma immunotherapy analysis and drug screening

**FIGURE 9:**
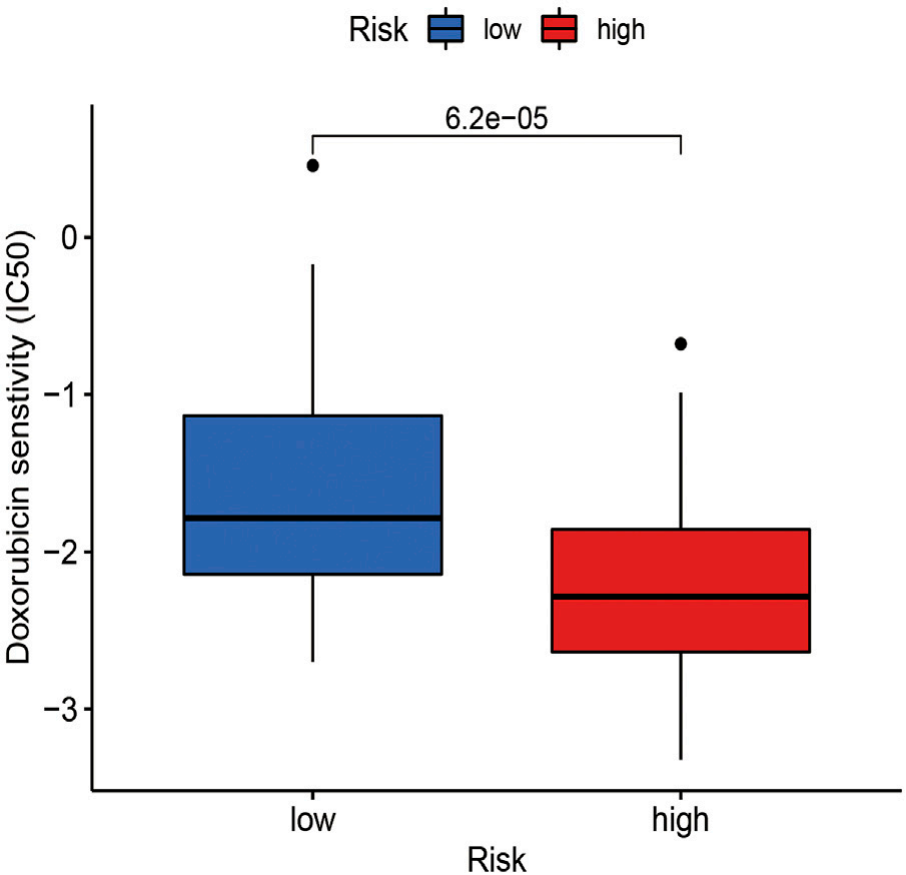
The estimated IC50 for doxorubicin was displayed for high- and low-risk groups in OSCC.

To better understand the individualized treatment of OSCC with drugs, this study used the drug analysis of the pRRophetic package to treat OSCC to obtain anticancer drugs sensitive to OSCC. A significant difference in doxorubicin sensitivity was seen between the high-risk and low-risk groups, as shown by Figure 9. This finding indicates that doxorubicin has a potential effect in the treatment of OSCC.

### Quantitative real-time PCR validation of long non-coding RNAs

**FIGURE 10:**
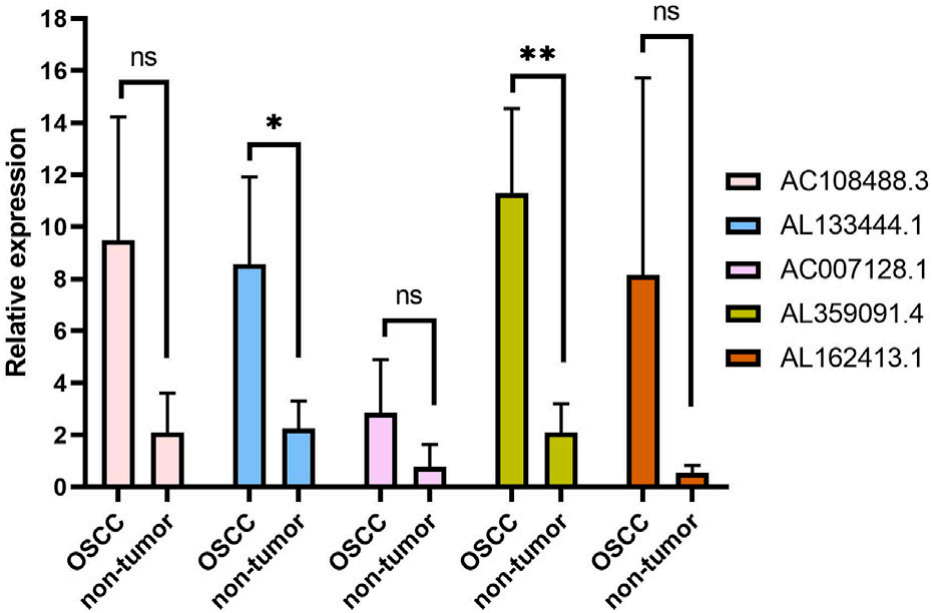
The qPCR analysis of five lncRNAs related to the m7G gene in normal tissue and OSCC. *p < 0.05, **p < 0.01 and ns No significance.

To further verify the expression levels of five m7G-related lncRNAs in nontumor oral tissues and OSCC tissues, the expression levels of the five lncRNAs in OSCC tissues all showed an upwards trend after RT‒qPCR (Figure 10). Further statistical analysis, however, indicated that only AL133444.1 and AL359091.4 showed significant differences between nontumor tissues and OSCC. The other three lncRNAs (AC108488.3, AC007128.1 and AL162413.1) did not show a statistically significant difference. This should be attributed to the small sample size in this study.

## Discussion

However, the prognosis of OSCC patients has been improved to some extent after surgery and chemotherapy. However, for advanced and metastatic OSCC patients, their prognosis is not rational. Therefore, it is urgent for OSCC patients to explore new prognostic markers. Additionally, lncRNAs have been linked to the development and treatment of cancer through biological mechanisms. Research has demonstrated that m7G-related genes play an important role in cancer development. Since then, m7G-related lncRNAs have been increasingly used in tumor research, but there are few reports on m7G-related lncRNAs in OSSC. Therefore, the potential interaction between m7G and lncRNAs should arouse our attention and reveal potential prognostic markers.

In this study, the m7G methylation complex NCBP2, NUDT4, EIF4E3, LARP1 and EIF4E2 were used to determine the lncRNAs related to prognosis. Then, five lncRNAs (AC108488.3, AL133444.1, AC007128.1, AL359091.4 and AL162413.1) were used to construct new prognostic signals of OSCC. Although some of these five lncRNAs have been reported in tumor research, there is still a lack of research in OSCC. Liu H et al. reported that the prognostic signal constructed by AC007128.1 and seven other lncRNAs can predict the prognosis of esophageal cancer patients. In addition, lncRNA AC0071288.1 was found to be upregulated in cells as well as tissues of esophageal squamous cell carcinoma, which is closely related to the poor prognosis of these patients (Zhang et al., 2021). Moreover, AC007128.1 can activate the MAPK/ERK and MAPK/p38 pathways in ESCC cells, leading to epithelial-mesenchymal transformation (Zhang et al., 2021). Lu et al. identified four pyroptosis-related lncRNAs by multivariate Cox regression analysis, including AC007128.1, thus constructing a prognostic risk model (Lu et al., 2022). Using this model, individualized risk assessments can be conducted and clinical treatment recommendations can be provided based on the level of lncRNA pyrophosphate. Using five m7G-related lncRNAs, a prognostic risk model was constructed that also provides insight into OSCC prognosis and treatment. Our study focused on RT‒PCR analysis of five lncRNAs from clinical samples. Using a small sample size, only AL133444.1 and AL359091.4 differed from nontumor samples in OSCC. Furthermore, we found that there were large differences in the expression levels of lnRNAs across samples in the same group. It is therefore necessary to conduct further clinical research on expanded samples to confirm the expression levels of these five lncRNAs in OSCC.

As an important parameter of tumor clinical pathology, the American Joint Commission on Cancer (AJCC) staging system is widely used in tumor evaluation (Giannis et al., 2021). The clinical features associated with tumor grade were not different among groups based on a risk score constructed from five m7G-related lncRNAs. It is important to note that the results above are based on a small number of possible patients with different tumor grades. However, there were significant differences between OSCC patients’ living conditions. Based on m7G-related lncRNAs, this result was in agreement with the prognosis of high- and low-risk OSCC patients. Compared with TNM staging, the 1-, 3- and 5-year prognosis AUC of the constructed risk signal is greater than 0.7, which indicates that the prognostic signal constructed by lncRNAs also has certain advantages. In this study, the prognostic model of the nomogram was constructed by multivariate logistic regression, and its combined factors included five lncRNAs, stage T and stage N. Calibration curves show that the nomogram is reliable for predicting and judging OSCC prognoses. At the same time, the C-index curve is above that of other pathological staging systems, which further indicates the advantages of the nomogram. In comparison with the OSCC prognosis model constructed by currently known lncRNAs, the evaluation index c-index of seven pyroptosis-related long noncoding RNAs in model construction was 0.6 (Li et al., 2017). We found some advantages with OSCC prognosis models based on m7G-related lncRNAs in our research. One such advantage was a C-index greater than 0.7 for 1–5-year prognosis predictions. Although DCA is widely used in tumor prognosis decision-making, the model of m7G-related lncRNAs is seldom used in tumor prognosis decision-making, and OSCC has never been reported. In this study, the DCA curve shows that the nomogram has certain value in clinical decision-making because the nomogram curve is higher than that of other pathological staging systems in a certain range.

During tumor growth and progression, the immune system plays a critical role. Among them, the existence of tumor-infiltrating lymphocytes is related to the improvement of the survival rate of head and neck squamous cell carcinoma (Almangush et al., 2020). CD8^+^ T-cell tumor invasion is significantly associated with better prognosis in HNSCC patients compared to patients without invasion. These findings are consistent with those of this study. Regarding the immune infiltration of OSCC, the score of CD8^+^ T cells in the high-risk group was lower than that in the low-risk group (Ogino et al., 2006). Therefore, CD8 is expected to be an important parameter to evaluate the prognosis of OSCC. Shimizu et al. reported that in patients with OSCC, CD8^+^ T cells can provide an evaluation index for tumor recurrence and prognosis in the invasion margin and surrounding stroma (Shimizu et al., 2019). Gu X et al. compared with the normal tongues of healthy people and found that the cytolytic activity of SCCOT patients was increased (Gu et al., 2019). In this paper, we found that patients with OSCC have higher cytolytic activity at high risk. Similar findings were reported by Tang Y et al. in a study that compared high-risk and low-risk HLA for head and neck cancer based on iron death-related lncRNAs (Tang et al., 2021).

In the prediction of chemotherapy drugs for OSCC patients based on m7G-related lncRNAs, doxorubicin’s IC50 was lower in the high-risk group. Based on this, the high-risk group exhibits more sensitivity to doxorubicin, which will provide a reference for the high-risk group. Doxorubicin is associated with nausea, vomiting, and blood cell dysfunction in OSCC patients. To improve the curative effect of doxorubicin, the dosage form is often modified (Moradzadeh Khiavi et al., 2019). Li et al. reported that doxorubicin encapsulated in biodegradable micelles degraded faster in tumors than in normal tissue (Li et al., 2017). Furthermore, this dosage form induces local sustained release of the active ingredient and inhibits tumor growth in OSCC, but the organ does not suffer any damage resulting from the treatment (Li et al., 2017). Laser near-infrared light was also found to regulate drug release behavior in doxorubicin after microneedle assembly was discovered (Xu et al., 2020). With this innovative system, tumors can be eliminated, and side effects can be minimized, making it a powerful method for treating OSCC clinically. It is therefore important to increase the curative effectiveness of drug therapy by changing the dosage form of doxorubicin for patients with OSCC.

A number of limitations were identified in this study, despite the fact that it shows that lncRNAs derived from m7G are capable of predicting OSCC patient prognosis. First, the samples of the prediction model were only from TCGA, and the sample size was small. A second problem with this model is that there are no biochemical experiments to prove how it functions, so it is necessary to clarify how m7G-related lncRNAs regulate the OSCC pathological process.

## Conclusion

The aim of this study was to determine the best prognostic marker for OSCC patients based on five m7G-related lncRNAs. Therefore, we will provide a new idea and method for immunotherapy and individualized treatment of OSCC patients and reflect that five m7G-related lncRNAs may be therapeutic targets of OSCC in the future.

## Data Availability

The original contributions presented in the study are included in the article/Supplementary Material, further inquiries can be directed to the corresponding author.
