# Supplementary material for: m7G-related lncRNAs are potential biomarkers for predicting prognosis and immune responses in patients with oral squamous cell carcinoma
Source: Front Genet. 2022 Dec 2;13:1013312. doi: 10.3389/fgene.2022.1013312 (PMC9755667; doi:10.3389/fgene.2022.1013312)
Supplement: Supplementary file 1 [file Table1.DOC]

Table S1：The primer sequences of PCR

| GeneID | PrimerID | seq 5'-3' |
| --- | --- | --- |
| AC108488.3 | F | AACTCGTGCCGAATGATCCC |
|  | R | TGGGAATCCTGAGAGAGGGC |
| AL133444.1 | F | GCTAACTAATCAGGGCTGTGGA |
|  | R | TGGATGTTCTTGGCATCTCG |
| AC007128.1 | F | GGACAAGCGCACAAAAGGTT |
|  | R | AGTGGCACTTCGCACTACTC |
| AL359091.4 | F | GGGACCAGAGCGGGATTATG |
|  | R | CACGAGGCACCTCTCAAAGT |
| AL162413.1 | F | AGAAAGCCATCATTCTACCATCAA |
|  | R | GGGACATTCAGTTTTCTCTTTGC |
